# Osteopontin Is Expressed in the Mouse Uterus during Early Pregnancy and Promotes Mouse Blastocyst Attachment and Invasion *In Vitro*

**DOI:** 10.1371/journal.pone.0104955

**Published:** 2014-08-18

**Authors:** Qian-Rong Qi, Qing-Zhen Xie, Xue-Li Liu, Yun Zhou

**Affiliations:** 1 Center for Reproductive Medicine, Renmin Hospital of Wuhan University, Wuhan, P. R. China; 2 Medical College of Wuhan University, Wuhan, P. R. China; State Key Laboratory of Reproductive Biology, Institute of Zoology, Chinese Academy of Sciences, China

## Abstract

Embryo implantation into the maternal uterus is a decisive step for successful mammalian pregnancy. Osteopontin (OPN) is a member of the small integrin-binding ligand N-linked glycoprotein family and participates in cell adhesion and invasion. In this study, we showed that *Opn* mRNA levels are up-regulated in the mouse uterus on day 4 and at the implantation sites on days 5 and 8 of pregnancy. Immunohistochemistry localized the OPN protein to the glandular epithelium on day 4 and to the decidual zone on day 8 of pregnancy. OPN mRNA and proteins are induced by *in vivo* and *in vitro* decidualization. OPN expression in the endometrial stromal cells is regulated by progesterone, a key regulator during decidualization. As a secreted protein, the protein level of OPN in the uterine cavity is enriched on day 4, and *in vitro* embryo culturing has indicated that OPN can facilitate blastocyst hatching and adhesion. Knockdown of OPN attenuates the adhesion and invasion of blastocysts in mouse endometrial stromal cells by suppressing the expression and enzymatic activity of matrix metalloproteinase-9 in the trophoblast. Our data indicated that OPN expression in the mouse uterus during early pregnancy is essential for blastocyst hatching and adhesion and that the knockdown of OPN in mouse endometrial stroma cells could lead to a restrained *in vitro* trophoblast invasion.

## Introduction

In placental mammals, the implantation of an embryo into the maternal uterus is a pivotal step for the successful establishment of pregnancy and is likely to be mediated by a series of signaling and adhesion molecules. Embryo implantation begins at the interaction between the uterine luminal epithelium (LE) and the hatched blastocyst. After the adherence of the trophoderm to luminal epithelial cells, trophoblast cells invade the uterine stroma and uterine stromal cells undergo decidualization, which is characterized by extensive proliferation and differentiation and contributes to placentation and pregnancy maintenance [1]. In mice, the uterus is receptive to blastocysts during a spatiotemporally restricted time termed the “implantation window”, during which the blastocyst hatches from the zona pellucida and attaches to the uterine epithelium at day 4.5 of pregnancy. In humans, implantation occurring in a non-receptive uterus or beyond the implantation window results in infertility or spontaneous abortions [2]. Functional studies using animal models and Large-scale Sequencing Research have revealed a number of molecules that function in implantation and decidualization [3], including leukemia inhibitory factor (LIF) [4], interleukin 11 [5] and epidermal growth factor receptor [6]. However, the molecular basis underlying implantation and decidualization remains poorly understood.

Previous studies identified a number of molecules that have been shown to peak during the window of implantation, including, LIF [7], integrin αvβ3 [8] and fibronectin [9]. Integrins are cell adhesion receptors belonging to the integrin family that function by recognizing multiple ligands, including laminin [9], collagen [10], epiligrin [11], and vascular cell adhesion molecule [12]. The major integrin binding site is an Arg-Gly-Asp (RGD) tripeptide present in a variety of integrin ligands. Contact regions for the RGD sequence have been identified in the integrin subunits. Osteopontin (OPN), a member of the small integrin-binding ligand N-linked glycoprotein family, is able to bind to cell surface integrins through its RGD sequence, promoting cell adhesion and invasion [13]. The OPN protein is expressed at a high level in the uterine epithelium during the mid-secretory phase, in the decidua and in the cytotrophoblast in humans [14]. Studies in sheep and swine have indicated that OPN is involved in the interaction between uterine LE and the trophectoderm [15], [16]. In mice, OPN is expressed in the uterine glandular epithelium (GE) on day 4 [17] and in the immune cells surrounding the decidual cells during early pregnancy [18]. OPN-deficient mice manifested a decreased pregnancy rate during mid-gestation compared to wild-type mice, suggesting peri-implantation pregnancy loss [19]. OPN expression in the peri-implantation period may be involved in blastocyst implantation and decidualization.

Stromal decidualization is a critical process that enables correct trophoblast invasion and placenta formation, which are regulated by both the trophoblast and the stroma [20]. Matrix metalloproteinases (MMPs) are a group of extracellular matrix (ECM) proteases involved in tissue remodeling in both physiological and pathophysiological conditions, including decidualization and placentation [21]. The invasion of the trophoblast into stroma depends on the embryo-secreted proteinases, which degrade the ECM components [22]. MMP-9 is the predominant MMP secreted from activated blastocysts, and its expression begins around Day 6, when the blastocysts begin to invade the maternal stroma [23]. Published results suggest that OPN could induce the expression of MMP-9 in some cell types to mediate cell invasion in tumorigenesis [24], [25]. Whether OPN secreted from the endometrium is able to promote trophoblast invasion by up-regulating the expression of MMP-9 is still poorly understood.

Our objective is to examine the function of OPN during early pregnancy in mice and its effect on trophoblast invasion and outgrowth in mouse endometrial stromal cells (mESC). Here, we demonstrate that OPN expression in uterine GE on day 4 is regulated by estrogen, whereas the OPN expression in stromal cells is regulated by progesterone. Secreted OPN is able to facilitate blastocyst hatching and adhesion. Furthermore, OPN expression in the decidua is related to trophoblast invasion by regulating the expression and enzymatic activity of MMP-9.

## Materials and Methods

### Animal treatments

Sexually mature mice (Kunming White outbred strain, 6–8 weeks) were maintained in a controlled environment (14 h light and 10 h dark cycle). All animal procedures were approved by the Institutional Animal Care and Use Committee of Wuhan University.

Female mice were super-ovulated by an injection of 7.5 IU of PMSG (Lizhu Company, Zhuhai, China), followed by 7.5 IU of hCG (Lizhu Company, Zhuhai, China) 48 h later. After the hCG injection, the mice were mated with fertile or vasectomized males of the same strain to induce normal pregnancy or pseudopregnancy (day 1 = the day of the vaginal plug). For uterine horn ligation treatment, one uterine horn was ligatured by 6–0 silk thread at the joint part of uteri-cervix under chloral hydrate anesthesia, then the female mice were mated with fertile males after a week later. From days 1 to 4, pregnancy was confirmed by flushing the embryos from the oviducts or uteri. The implantation sites on day 5 were visualized through intravenous injections of 0.1 ml of 1% Chicago blue dye (Sigma-Aldrich Inc., St. Louis, MO, USA) in saline. The pregnant uteri at different times were fixed for immunohistochemistry or collected for RNA and protein extraction. Artificial decidualization was induced by intraluminal injections of 10 µl of sesame oil (Sigma-Aldrich Inc., St. Louis, MO, USA) into one uterine horn on day 4 of the pseudopregnancy, using the contralateral uterine horn as control. The uteri were collected on day 8 of pseudopregnancy.

Steroid hormonal treatments were initiated 2 weeks after ovariectomy. Ovariectomized mice were injected subcutaneously with estradiol-17β (100 ng/mouse, Sigma), progesterone (1 mg/mouse, Sigma), or estradiol-17β plus with progesterone, whereas the control group was only injected with sesame oil (Sigma). For ICI 182,780 treatment, ovariectomized mice were injected with ICI 182,780 (1 mg/mouse, AstraZeneca, London, UK) an hour before estradiol-17β injection. Mice were sacrificed 24 h after hormone injections, and their uteri were collected for RNA and protein analysis.

### Uterine flushing fluids collection

From day 3 to 5 of pregnancy, the uterine flushing fluids were collected by flushing the uterine horns with 200 µl of saline water. The liquids were centrifuged to discard hemocytes, castoff cells and embryos; the supernatant was collected; and the proteins were extracted by TCA-Acetone precipitation methods, as previously described [26]. The extracted protein concentration was detected by a BCA reagent kit (Applygen, Beijing, China), diluted to a unified concentration and used for western blot analysis.

### Embryo collection and culture

Mouse blastocysts were collected at 08:00 on day 4 of pregnancy, and the zona pellucida (ZP) was examined under a microscope before being transferred into single-step medium (Irvine, USA) under mineral oil (Sigma) and cultured at 37°C, 5% CO_2_ with BSA, different concentrations of recombinant mouse OPN protein (rOPN, R&D Systems, Minneapolis, USA, 0.1 µg/mL, 1.0 µg/mL and 10.0 µg/mL), goat IgG (Santa Cruz Biotechnology) or anti-OPN antibodies (Santa Cruz Biotechnology, Santa Cruz, CA, 0.01 µg/mL, 0.1 µg/mL and 1.00 µg/mL). Fourteen hours later, the hatching rate was examined by three independent persons, and the experiments were repeated three times.

### Embryo adhesion assay

The 96-well plates were pre-coated with fibronectin (FN, 1 mg/mL, Sigma) at 37°C and 5% CO_2_ for 2 h. The 25 hatched blastocysts were seeded into each well in 50 µl of single step medium with 10.0 µg/mL of rOPN, 1.00 µg/mL of anti-OPN antibody or 0.50 mg/mL of RGD peptide (Sigma), and BSA was used for the controls. Twenty-four hours later, the adhesion rate of the blastocysts was examined by three independent persons, and the experiments were repeated three times.

### Primary mESC culture and treatment

mESC were isolated as previously described [27]. Cells were cultured in complete medium consisting of DMEM-nutrient mixture F-12 Ham (DMEM/F-12, Sigma) with 10% charcoal-treated fetal bovine serum (cFBS, Life Technologies, California, USA) at a concentration of 5×10^5^ cells/dish or 2×10^5^ cells/well for 12-well culture plates.

*In vitro* decidualization was performed as described previously [27]. Briefly, mESC isolated from day 4 of pregnancy were treated with 10 nM estradiol-17β (Sigma) and 1 µM progesterone (Sigma), and the mESC were collected 24 h, 48 h and 72 h later. Mouse decidual prolactin-related protein (*Dtprp*) was detected using real-time PCR as a reliable decidualization marker.

The mESCs isolated from day 4 of pregnancy were treated with 10 nM estradiol-17β (Sigma), 1 µM progesterone (Sigma), or a combination of 10 nM estradiol-17β and 1 µM progesterone. The mESCs were collected 24 h later for further study.

The siRNAs targeting OPN were designed and synthesized by Ribobio Co., Ltd. (Guangzhou, China). The siRNA sequence targeting mouse OPN is GUCAGCUGGAUGAACCAAGUU. Both OPN-targeting siRNA and negative controls were transfected into cultured mESC with Lipofectamine 2000 following the manufacturer's instructions (Invitrogen). The transfection medium was replaced with DMEM/F12 containing 10% cFBS 6 h later, and the cells were harvested 24 h or 72 h for RNA and western blot analysis to detect the transfection efficiency.

### Immunohistochemistry

Formalin-fixed and paraffin-embedded sections were incubated with a mouse monoclonal OPN antibody (1∶200 dilution, Santa Cruz) or rabbit Ig G (1∶200 dilution, Santa Cruz) at 4°C overnight, respectively. After washing in PBS, the sections were incubated with HRP-conjugated secondary antibodies (Vector Laboratories, Burlingame, CA, USA) for 45 minutes at room temperature. The color was developed with a DAB kit (Vector Laboratories). Positive signals of OPN were visualized as brown in color. The data demonstrated in each figure were repeated at least three times.

### RNA extraction and real-time PCR

Total RNAs from the mouse tissues and cultured cells were extracted by a mRNA Capture Kit (Boehringer Mannheim, Mannheim, Germany) and reverse transcribed into cDNA with the PrimeScript reverse transcriptase reagent kit (TaKaRa Bio Inc., Tokyo, Japan). For real-time PCR, cDNA was amplified using a SYBR Premix Ex Taq kit (TaKaRa; DRR041S) on the Rotor-Gene 3000A system (Corbett Research, Mortlake, Australia) according to the manufacturer's recommendations. All reactions were run in triplicate. The corresponding primer sequences were used for real-time PCR. Gapdh served as an internal control. The primer sequences for *Opn* were 5-CACTCCAATCGTCCCTAC-3 and 5-AGACTCACCGCTCTTCAT-3; for *Dtprp*, 5-AGCCAGAAATCACTGCCACT-3 and 5-TGATCCATGCACCCATAAAA-3; and for *Gapdh*, 5-GTTGTCTCCTGCGACTTCA-3 and 5- GGTGGTCCAGGGTTTCTTA-3. Data from real-time PCR were analyzed by the 2^−△△Ct^ method. The significance of differences between the two groups was assessed by Student's t-test. Multiple comparisons were performed with Tukey's ANOVA. *p*<0.05 was considered statistically significant.

### Western Blot

Western blots were run as previously reported [28]. Samples were incubated with primary antibodies for OPN (Biorbyt, California, USA) or GAPDH (Cell Signaling Technology, Boston, USA) and then with matched second antibodies conjugated with horseradish peroxidase. The signals were developed with a ECL chemiluminescent kit (Amersham Biosciences, Boston, USA). All experiments were repeated three times.

### Embryo-mESC co-culture model and immunofluorescence

Two-cell embryos were collected at day 2 of pregnancy and cultured in single-step medium for another 2-day period. Hatched blastocysts were added to the confluent monolayers of OPN-targeted siRNA or NC-siRNA pre-treated mESC in DMEM/F12 containing 10% cFBS for 48 h. Immunofluorescence was performed on this embryo-mESC co-culture as previously described [29]. Cells were incubated with a rabbit E-cadherin antibody (1∶100 dilution, Cell Signaling Technology, Boston, USA), goat polyclonal MMP-9 antibody (1∶100 dilution, Santa Cruz) or rabbit Ig G (1∶100 dilution, Santa Cruz), followed by the FITC conjugated secondary antibody (PIERCE, Rockford, IL, USA). Nuclei were stained with DAPI (Zhongshan Golden Bridge Bio-technology, Beijing, China). The trophoblast spreading area was quantified by measuring the E-cadherin signal area using the Image J software. A total of 10 embryos were calculated in each experiment, and the experiments were repeated three times.

### Gelatin zymography

The gelatin zymography was performed as previously described [30]. The culture medium from the embryo-mESC co-culture model was mixed with the same volume of loading buffer (0.25 M Tris, 40% Glycerol, 8% SDS and 0.04% bromophenol blue) and incubated at 37°C for 30 min. The samples were loaded onto a 10% separating gel containing 1 mg/ml gelatin. After electrophoresis, the gels were washed with 2.5% Triton X-100 buffer (0.05 M Tris, 2.5% Triton, pH7.5) for 1 h and then incubated with 50 mM Tris–HCl, 200 mM NaCl and 5 mM CaCl_2_ pH 7.5 at 37°C overnight. Each was stained with Coomassie Blue R-250 for 1 h and destained in a solution containing 30% methanol and 10% acetic acid. The gel was scanned by an ultraviolet imaging system (Bio-RAD).

## Results

### OPN expression in the mouse uterus during early pregnancy

**Figure 1:**
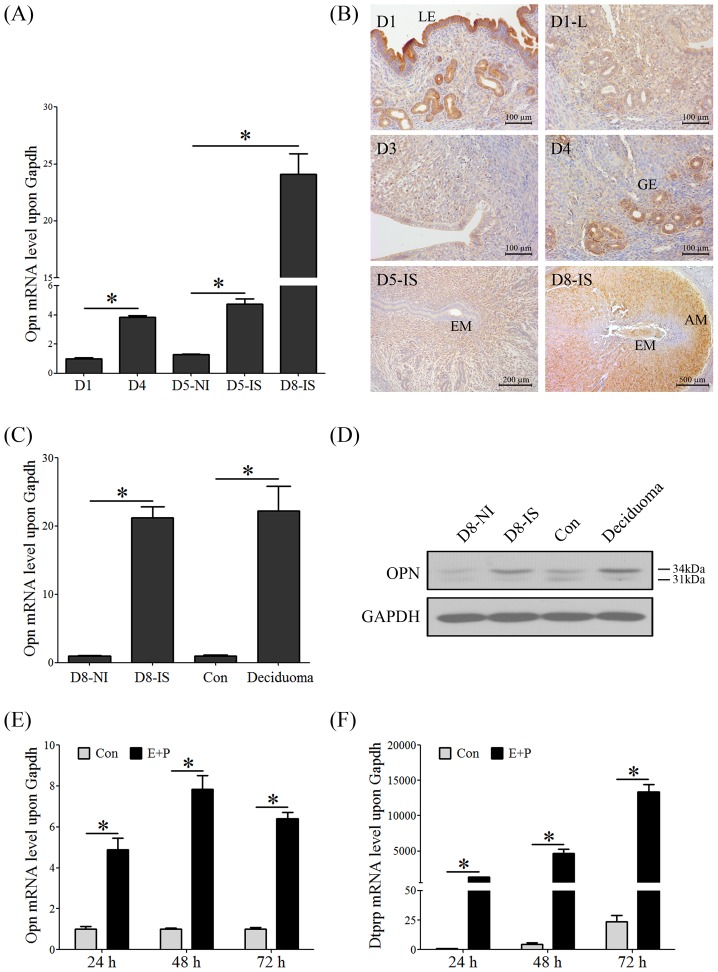
Expression of OPN in the mouse uterus during early pregnancy. (A) Real-time PCR was performed to quantify the *Opn* mRNA levels in the mouse uterus during early pregnancy (D1: day 1, D4: day 4, D5-NI: inter-implantation sites on day 5, D5-IS: implantation sites on day 5, D8-IS: implantation sites on day 8). (B) Immunohistochemistry was used to detect the OPN protein localizations in the mouse uterus during early pregnancy (D1-L: ligated uterine horn on day 1 of pregnancy, LE: luminal epithelium, GE: glandular epithelium, EM: embryo, AM: anti- mesometrium). (C) *Opn* mRNA level in the mouse uterus on day 8 of pregnancy and artificial decidualization. (D) OPN protein level in the mouse uterus on day 8 of pregnancy and artificial decidualization. (E) *Opn* mRNA level during the *in vitro* decidualization of mESC. (F) *Dtprp* mRNA level during the *in vitro* decidualization of mESC. *, *p*<0.05; *error bars,* S.E. All of the experiments were repeated three times.

Real-time PCR was performed to quantify the mRNA expression of *Opn* in the mouse uterus during early pregnancy. The level of *Opn* mRNA on day 4 of pregnancy was up-regulated compared to that on day 1 of pregnancy. In addition, the level of *Opn* mRNA at the implantation sites on day 5 and 8 was up-regulated compared with the inter-implantation sites on day 5 (Fig. 1A). Immunohistochemistry was used to identify the localization of the OPN protein in mouse uterus during early pregnancy. OPN protein signals were highly detected in the luminal epithelium on day 1 and gradually decreased on days 2 and 3, which is inconsistent with mRNA level, we considered the OPN protein in luminal epithelium is semen-originated. Uterine horn ligation was performed to eliminate the interference of semen, the results from ligated uterine horn showed OPN signal is much weaker than normal mice (Fig. 1B), indicating that the OPN protein in the luminal epithelium on day 1 of pregnancy is semen-originated. On day 4, the OPN protein signal was exclusively detected in the glandular epithelium. On day 5, the OPN protein signal was weakly detected in the subluminal stroma surrounding the implantation blastocyst, and on day 8, OPN protein was localized in the mesometrial region of the decidua and anti-mesometrial region of the vascular endothelial cells (Fig. 1B).

*Opn* mRNA was up-regulated in the mouse uterus, and OPN protein was mainly localized in the decidual zone on day 8 of pregnancy. Both the mRNA and protein levels of OPN were highly expressed at the implantation sites and deciduoma on day 8 of the normal pregnancy or artificial decidualization model (Fig. 1C and D, respectively). *In vitro* decidualization was assessed to investigate the *Opn* expression in the mESC. *Dtprp*, a reliable marker of mouse decidualization, was up-regulated by *in vitro* decidualization. Real-time PCR showed that the mRNA levels of *Opn* and *Dtprp* were significantly induced by *in vitro* decidualization (Fig. 1E and F).

### Regulation of ovarian steroid hormones over OPN expression

**Figure 2:**
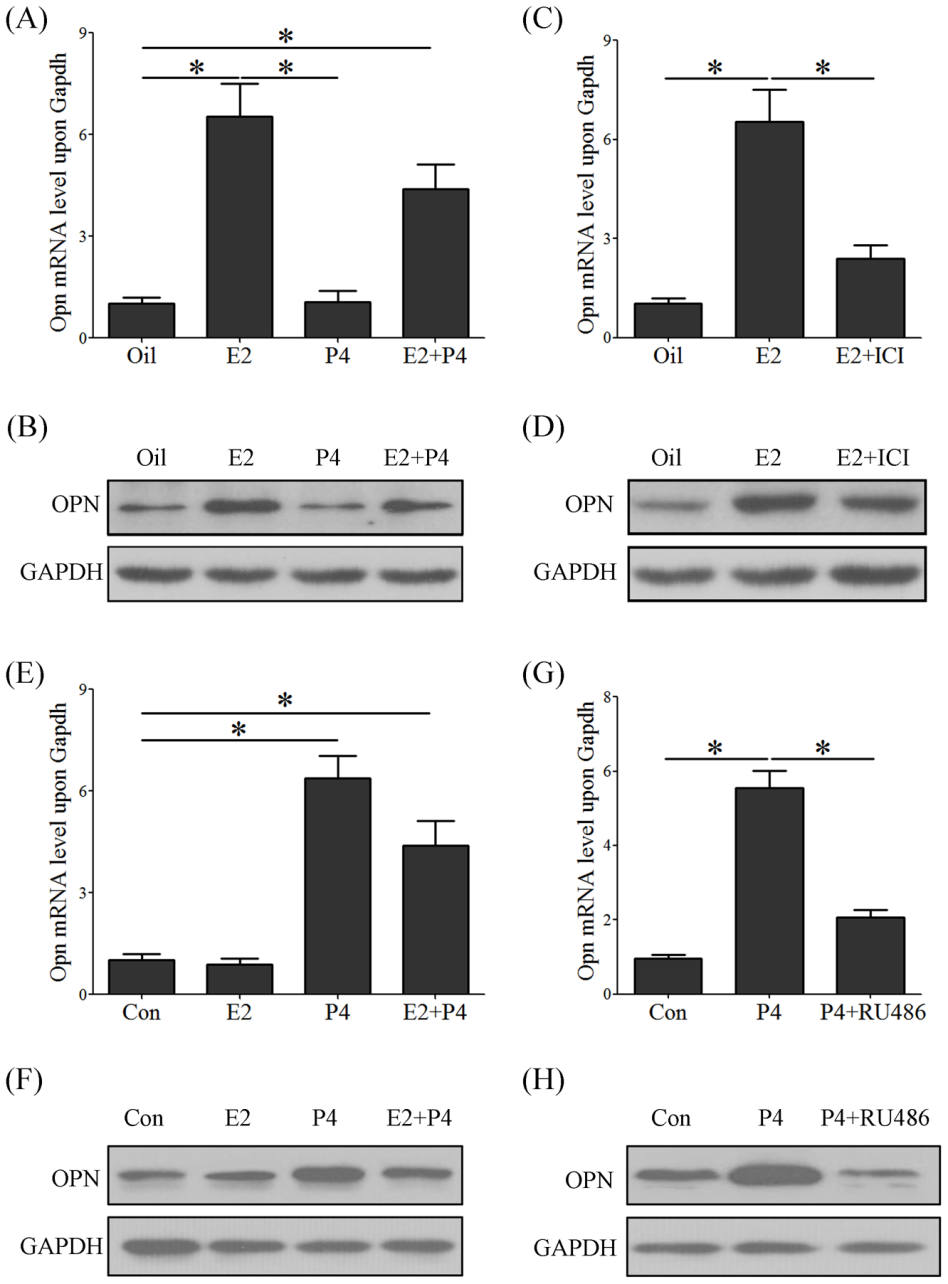
Ovarian steroids regulate OPN expression *in vivo* and *in vitro*. (A) Real-time PCR of *Opn* mRNA expression in ovariectomized mouse uteruses after hormone injections (Oil: sesame oil, E2: estradiol-17β, P4: progesterone, E2+P4: estradiol-17β plus with progesterone). (B) A western blot was performed to confirm the hormone regulation of OPN expression in mouse uteri; GAPDH was used as an internal reference. (C) Real-time PCR of *Opn* mRNA expression in ovariectomized mice uterus after the injection of estrogen alone or estrogen plus with ICI182,780, an estrogen receptor α antagonist (E2+ICI, estradiol-17β plus with ICI182,780). (D) A western blot was performed to confirm the hormone regulation on OPN expression in mouse uteri; GAPDH was used as an internal reference. (E) Real-time PCR of *Opn* mRNA levels after the mESCs were treated with steroids hormones. (F) Western blot analysis for the steroids hormones regulation of OPN expression in mESCs; GAPDH was used as an internal reference. G. Real-time PCR of *Opn* mRNA expression after the mESCs were treated with progesterone and RU486. (H) Western blot analysis for the progesterone regulation of OPN expression in mESCs, GAPDH was used as an internal reference. *, *p*<0.05; *error bars,* S.E. All of the experiments were repeated three times.

The estrogen secretion surge on day 4 of pregnancy is essential for mouse embryo implantation. Because *Opn* mRNA was highly expressed on day 4 of pregnancy, ovariectomized mice were used to examine the OPN expression in the mouse uterus, as regulated by ovarian estrogen or progesterone. Ovariectomized mice were treated with estrogen, progesterone or estrogen plus progesterone for 24 h. Data from real-time PCR showed that *Opn* mRNA was up-regulated in the estrogen (6.5-fold) and estrogen plus progesterone groups (4.4-fold) but not in the control or progesterone groups (Fig. 2A). Western blot results confirmed that the OPN protein is up-regulated in the estrogen group and in the estrogen plus progesterone group (Fig. 2B). To verify the regulation of estrogen on OPN expression, ICI 182,780, an antagonist that competes with estrogen for estrogen receptor α (ERα) binding, was used to examine whether estrogen regulates OPN through ERα. The up-regulation of the OPN mRNA and protein levels by estrogen could be impeded by ICI 182,780 injections (Fig. 2C and D).

Progesterone is the dominator regulator for stromal cell proliferation and differentiation during decidualization. OPN was highly expressed in the decidual zone and was mainly localized at the stromal cells. In primary cultured mESCs, the *Opn* mRNA and protein were significantly induced by progesterone (6.4-folds) or progesterone plus estrogen (4.1-folds), whereas estrogen had no effect on *Opn* expression in *in vitro* cultured mESCs (Fig. 2E and F). To further study progesterone regulation over OPN expression, RU486, a progesterone receptor antagonist, significantly abrogated progesterone-induced OPN expression (Fig. 2G and H).

### OPN expression facilitates blastocyst hatching and adhesion

**Figure 3:**
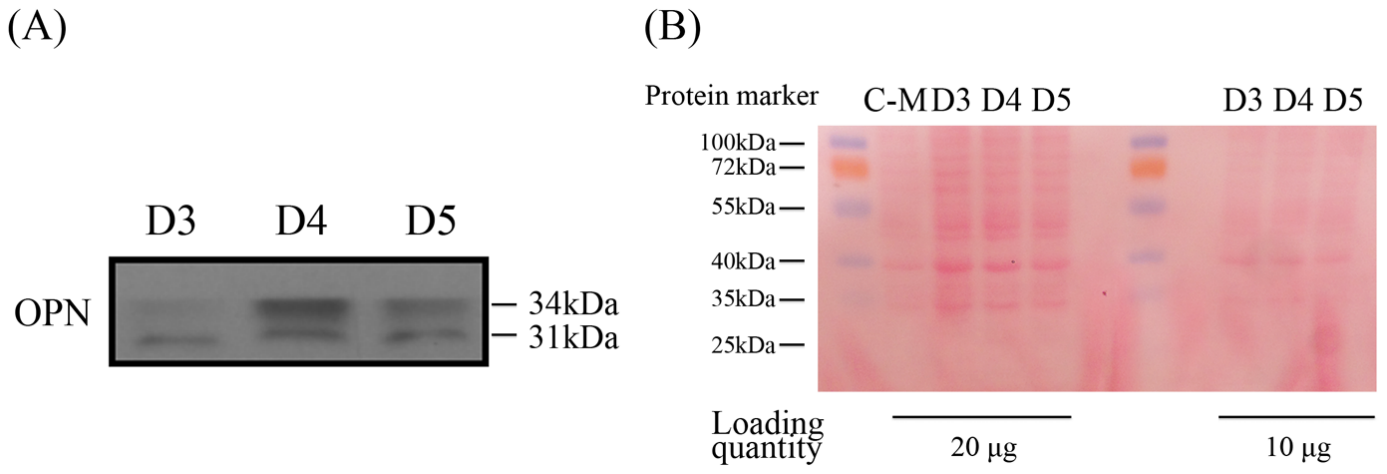
OPN protein level in uterine flushing fluids. (A) Western blot analysis for OPN protein expression levels in uterine flushing fluids. (B) Ponceau red staining of the western blot membranes (C-M: conditioned medium for embryo culture). All of the experiments were repeated three times.

Because the OPN protein was exclusively localized in the glandular epithelium on day 4 of pregnancy as a secreted protein, we deduced that OPN protein expression in the glandular epithelium was about to be secreted into the uterine cavity. Therefore, we collected uterine flushing fluids from day 3 to 5 of pregnancy and extracted the protein components. Western blots were performed to analyze the OPN protein levels in uterine cavity liquids during peri-implantation, and the results showed that the OPN protein was highly expressed in the uterine cavity liquids on days 4 and 5 of pregnancy, compared to day 3 (Fig. 3), suggesting that the OPN protein expressed in the glandular epithelium was mainly secreted into the uterine cavity and may play a role in maternal-embryo interactions during the implantation window. We also attempted to detect the OPN protein level in the embryo culture medium; however, the OPN protein level in the embryo culture medium was too low to detect.

**Figure 4:**
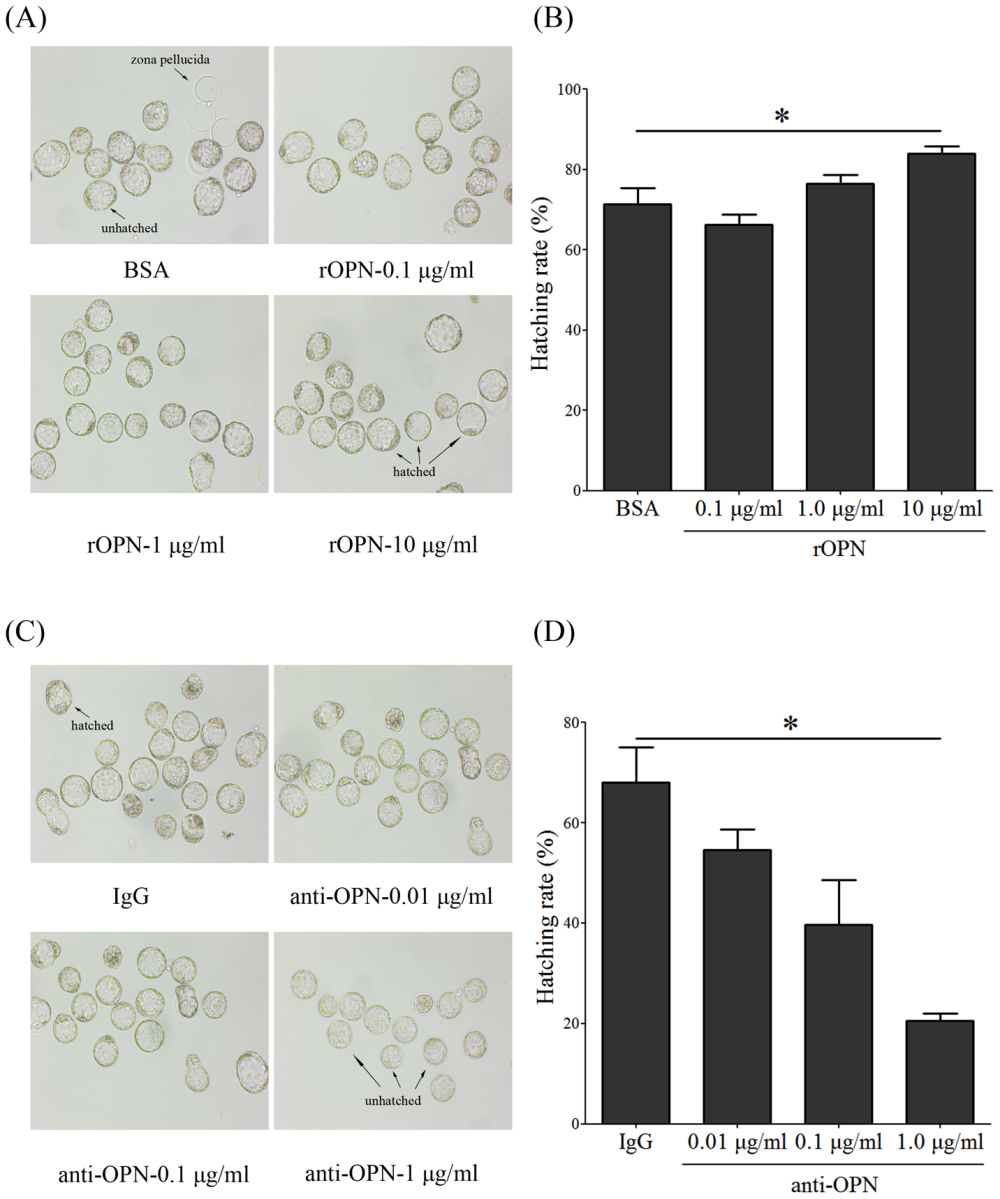
Blastocyst hatching assay. (A) Unhatched mouse blastocysts were collected at 08:00 on day 4 of the pregnancy and cultured with BSA (control) or rOPN at the concentrations of 0.1 µg/mL, 1.0 µg/mL or 10.0 µg/mL. (B) The hatching rate of blastocysts *in vitro* cultured with rOPN at concentrations of 0.1 µg/mL, 1.0 µg/mL or 10.0 µg/mL. *, *p*<0.05; *error bars*, S.E. (C) Unhatched mouse blastocysts were collected at 08:00 on day 4 of the pregnancy and cultured with IgG (control) and anti-OPN antibody at concentrations of 0.01 µg/mL, 0.1 µg/mL or 1.00 µg/mL. (D) The hatching rate of blastocysts *in vitro* cultured with anti-OPN antibodies at concentrations of 0.01 µg/mL, 0.1 µg/mL or 1.00 µg/mL. All of the experiments were repeated three times.

OPN is a secreted histotroph and adhesion molecule that is involved in cell-cell interactions. Our previous results demonstrated that the OPN protein exists in the uterine cavity's liquids on day 4 of pregnancy, and we hypothesized that OPN may participate in embryonic development and blastocyst adhesion during the implantation window. An *in vitro* embryo culture experiment was performed to examine the effect of OPN proteins on blastocyst hatching, and unhatched blastocysts were collected from day 4 and were cultured with rOPN at different concentrations of 0.1 µg/mL, 1.0 µg/mL and 10.0 µg/mL. Compared with the BSA treatment (71.%), the hatching rate of blastocysts was increased by the rOPN treatment at a concentration of 10.0 µg/mL (84.0%) (Fig. 4A and B). OPN binds to several ligands and mediates cell-cell interactions. Previous studies have confirmed that the OPN protein can be found in the mouse blastocyst and is involved in embryo activation by binding to integrins [17], [31]. We used anti-OPN antibodies to block the actions of secreted OPN on blastocyst activation. Compared with the IgG group, the hatching rates of the blastocysts were decreased by the anti-OPN antibodies at the concentrations of 0.01 µg/mL (54.7%), 0.1 µg/mL (40.0%) and 1.00 µg/mL (20.6%), respectively (Fig. 4C and D), indicating that intrauterine-located OPN proteins can promote blastocyst hatching.

**Figure 5:**
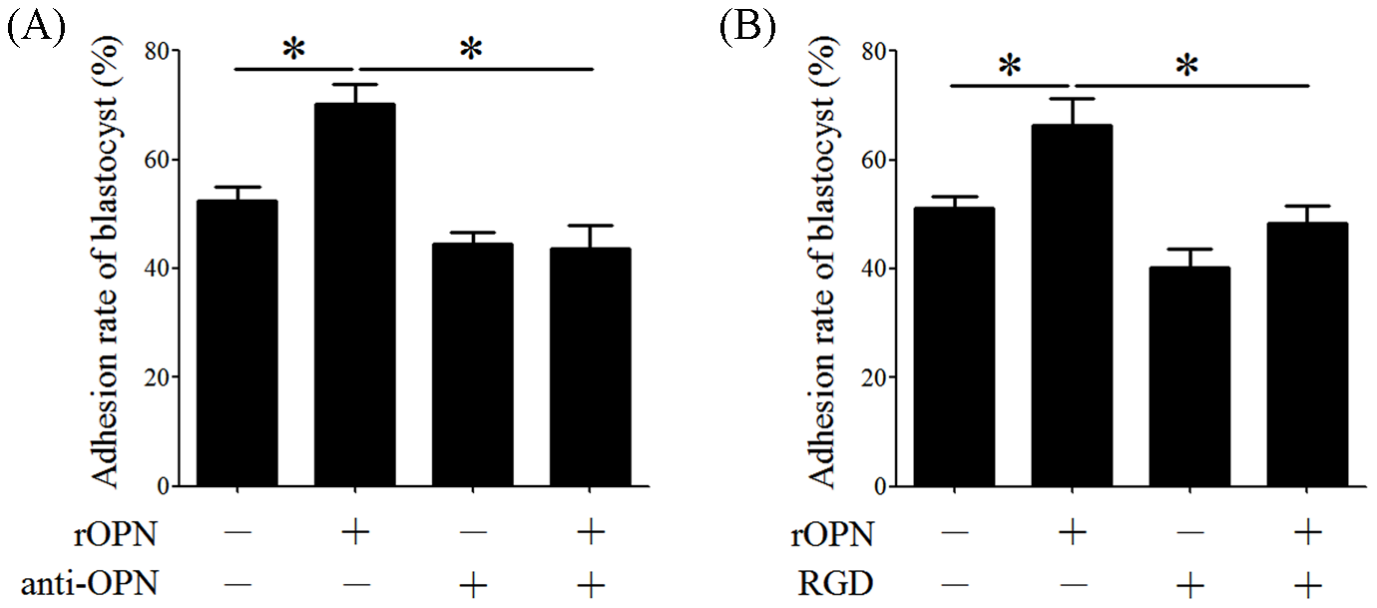
Blastocyst adhesion assay. (A) The adhesion rate of blastocysts in FN pre-coated dishes with BSA (control), rOPN or anti-OPN antibodies. (B) The adhesion rate of blastocysts in FN pre-coated dishes with BSA (control), rOPN or RGD peptides. *, *p*<0.05; *error bars,* S.E. All of the experiments were repeated three times.

OPN is an adhesion molecule and is highly expressed in the implantation window. Blastocyst adhesion assays were used to determine the function of OPN in blastocyst adhesion. The adhesion rate of blastocysts in rOPN-treated plates (70.3%) was higher than in the BSA-treated group (52.4%), whereas anti-OPN antibodies could neutralize the effects of rOPN on blastocyst adhesion (43.6%), indicating that OPN is able to facilitate blastocyst adhesion (Fig. 5A). Previous studies have revealed that OPN contains a RGD sequence that binds to integrins and mediates cell attachment. To determine whether the function of OPN on blastocyst adhesion occurs through the RGD sequence, soluble RGD peptides were added into FN pre-coated plates, and the results showed that he blastocyst adhesion rate was reduced by RGD peptides (48.3%) (Fig. 5B).

### OPN silencing limits trophoblast outgrowth and invasion *in vitro*

**Figure 6:**
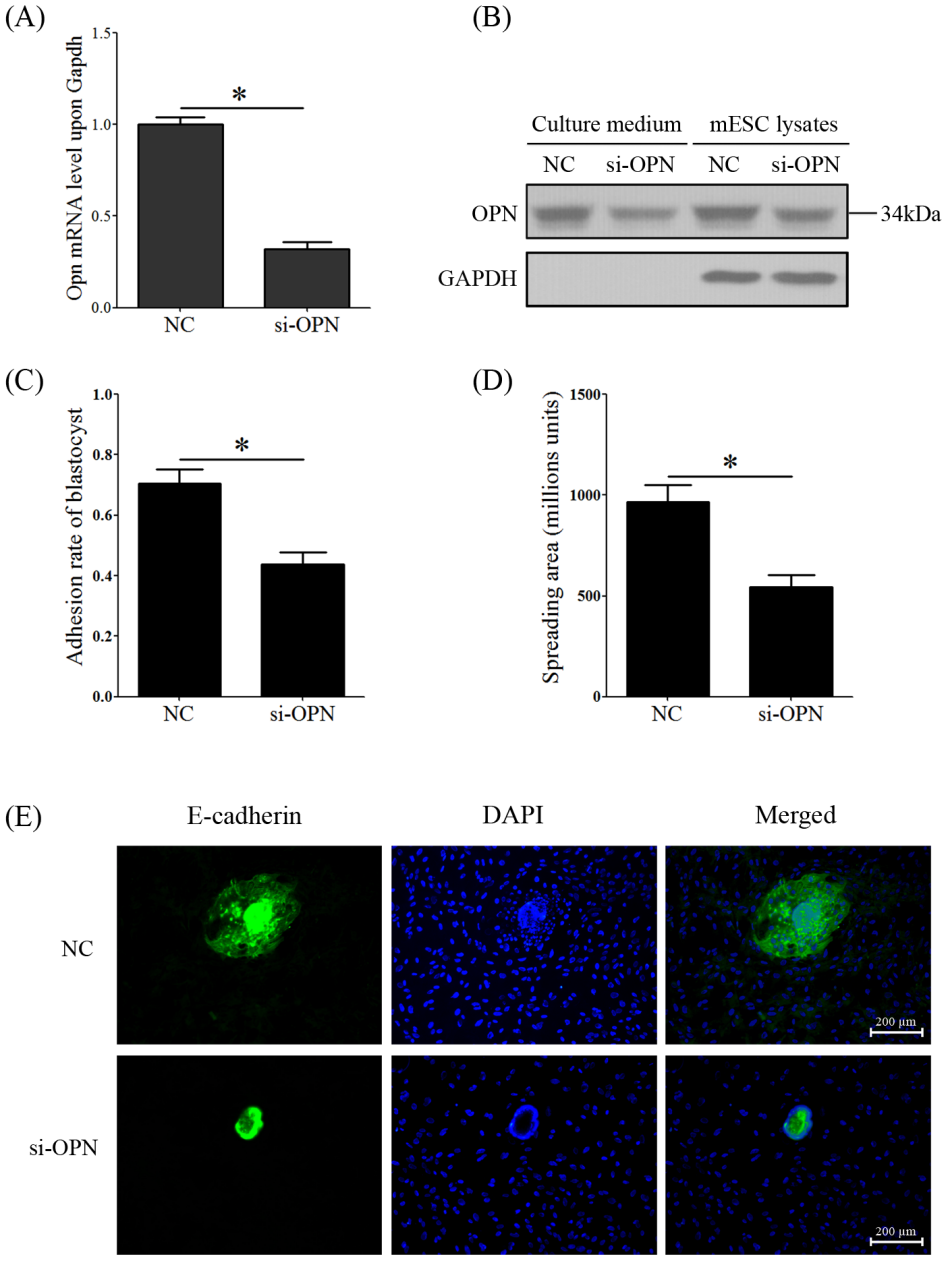
OPN silencing limits trophoblast outgrowth and invasion *in vitro*. (A) Real-time PCR of *Opn* mRNA levels in OPN-knockdown mESC by OPN siRNA transfection (NC: negative control siRNA, si-OPN: OPN siRNA). (B) A western blot confirmed the knockdown effect of OPN-targeted siRNA on OPN protein expression in mESCs or on secreted OPN proteins in the culture medium. (C) The adhesion rate of mouse blastocysts in control and OPN-knockdown mESCs. (D) OPN-targeted, siRNA-pretreated mESCs significantly decreased the trophoblast-spreading areas in the mESCs compared to the control group. The data for calculating the mean spreading area included at least ten embryos for each group, and all of the experiments were repeated at least three times. *, *p*<0.05; *error bars*, S.E. (E). Immunofluorescence was performed to detect the trophoblast outgrowth and invasion in OPN-targeted siRNA pretreated mESCs (Control, negative control siRNA pretreated mESC; si-OPN, OPN-targeted siRNA). Mouse embryos were marked by E-cadherin (Green signal), and nuclei were marked by DAPI (Blue). All of the experiments were repeated three times.

Because the OPN protein was expressed in the subluminal stroma surrounding the implantation blastocyst on day 5 and in the mesometrial and antimesometrial regions of the decidua on day 8, we hypothesized that OPN was involved in trophoblast invasion and tissue remodeling during decidualization. Real-time PCR confirmed that *Opn* mRNA was knocked down by OPN siRNA transfection in mESC, with an average knockdown efficacy of 68.1% (Fig. 6A). Western blots showed that the expression of secreted OPN proteins in the culture medium and OPN proteins in the mESC were both inhibited by OPN siRNA transfection (Fig. 6B). An *in vitro* co-culture model was used to investigate the adhesion and invasion of mouse embryos in OPN siRNA pre-treated with mESC. The hatched blastocysts were seeded on a confluent monolayer of OPN siRNA pre-treated mESC and cultured at 37°C and 5% CO_2_. Blastocyst adhesion in the OPN siRNA group was partially impeded compared to negative controls (Fig. 6C). Immunofluorescence was performed on the co-cultured mESCs and blastocysts to examine the trophoblast spreading area in the mESCs. We compared the trophoblast-spreading area by calculating the signal area of E-cadherin, a marker of trophoblasts, and found that the trophoblasts in the control mESCs displayed a more extensive spreading area than those in OPN siRNA-pretreated mESCs (Fig. 6D and E).

### OPN regulation on the expression of MMP-9 in trophoblast

**Figure 7:**
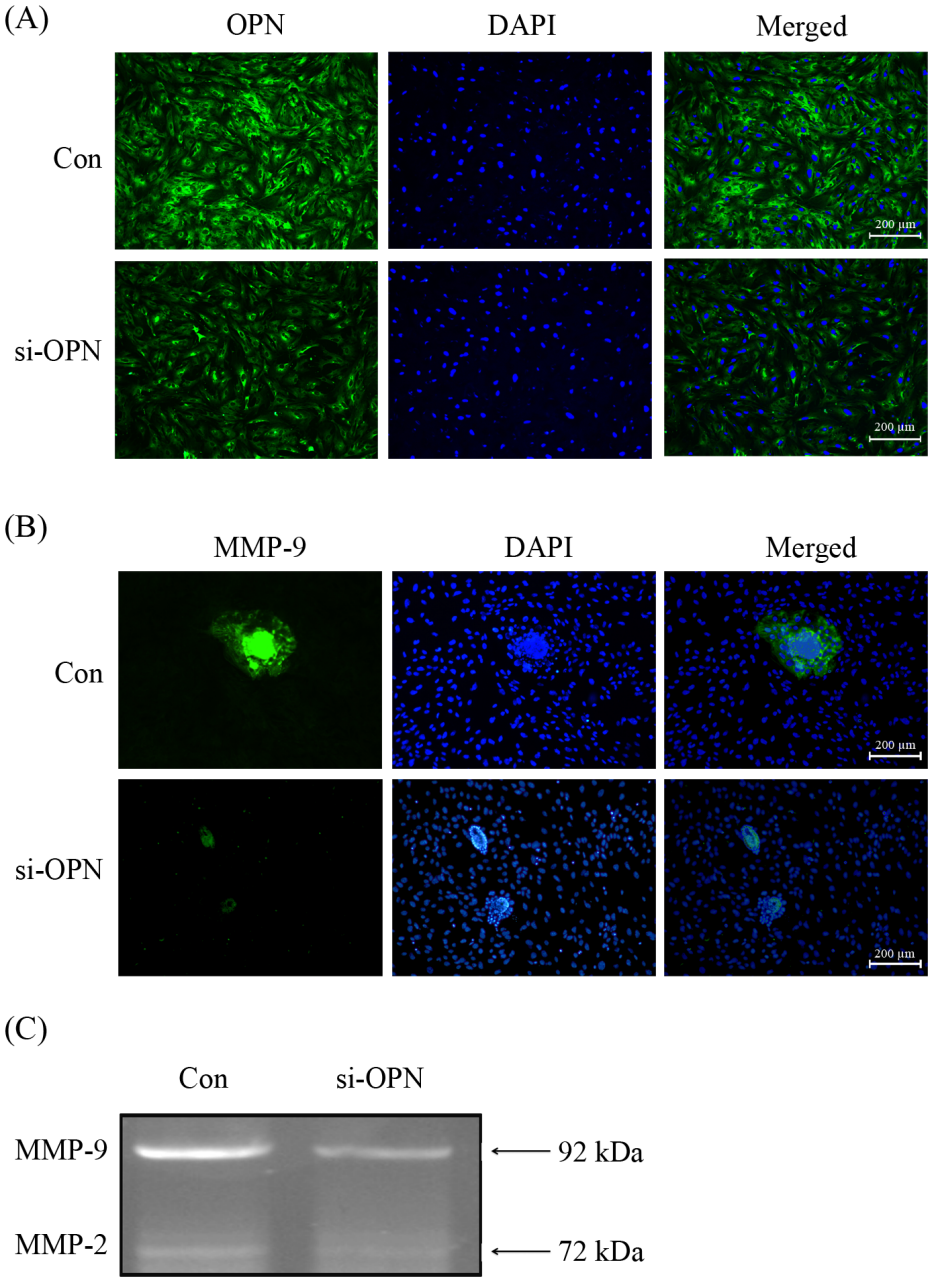
OPN regulates the expression and enzymatic activity of MMP-9 in the trophoblast. (A) Immunofluorescence was performed to detect the in situ OPN protein expression in OPN siRNA-pretreated or negative control siRNA-pretreated mESCs. (B) Immunofluorescence was performed to detect the trophoblast MMP-9 expression in OPN siRNA-pretreated or negative control siRNA-pretreated mESCs. Mouse embryos were marked by MMP-9 (Green signal), and nuclei were marked by DAPI (Blue). (C) Gelatin zymography was used to detect the enzymatic activity of MMP-9 and MMP-2 in the mESC-embryo culture medium, and MMP-9 enzymatic activity was inhibited by OPN silencing in the mESCs, when compared with negative controls. All of the experiments were repeated three times.

After finding that OPN knockdowns in mESCs can inhibit the invasion and outgrowth of trophoblast cells and that MMPs are involved in tissue remodeling during embryonic implantation into the maternal stroma, previous studies demonstrated that MMP-9 is a major facilitator of ECM degradation during implantation. We hypothesized that uterine-originating OPN proteins would be able to influence trophoblast invasive competence by regulating the expression of MMPs. The immunofluorescence results showed that the trophoblast expression of MMP-9 was significantly suppressed by OPN siRNA transfection in mESCs wen compared with negative controls (Fig. 7A). In addition, we collected the culture medium for gelatin zymography, and the results showed that the enzymatic activity of MMP-9 was reduced by OPN siRNA (Fig. 7B).

## Discussion

### The expression and regulation of OPN during early pregnancy

Embryo implantation is an intricate progress that requires an effective reciprocal interaction between a competent blastocyst and a receptive uterus. The implantation window of uterus is defined as the limited time when the uterine endometrium is receptive to blastocysts. Blastocyst activation is a pivotal step for successful implantation, during which the blastocyst hatches from the ZP and acquires adhesion and invasion capacities. OPN, also known as secreted phosphoprotein 1 (SPP1), is a member of the small integrin-binding ligand N-linked glycoprotein family and has been identified as playing an important role in both embryo implantation and the maintenance of pregnancy in sheep and pigs [31]. Further roles implicate tumorigenesis, tumor invasion and metastasis in humans [32]. In this study, compared with days 1 to 3 of pregnancy, the mRNA level of *Opn* is up-regulated on day 4, whereas the signal of the OPN protein on day 1 is much stronger than on day 4 of pregnancy and is mainly localized on the surface of the luminal epithelium. In situ hybridization has verified that *Opn* mRNA is primarily localized in GE and is weakly expressed in the LE on day 4 but not on day 1 [17]. In addition, OPN is present in semen and has been implicated in male reproduction [33], [34]. Our results from ligated uterine horn indicated that the OPN protein in the luminal epithelium on day 1 of pregnancy is semen-originated. For these reasons, we believe that the OPN protein localized in LE on day 1 originates from the semen and is possibly related to sperm capacitation and fertilization.

Estrogen (E2) and progesterone (P4) are superior regulators of reproduction, and the sequential and coordinated interplay between E2 and P4 maintains a normal reproductive cycle and pregnancy. In mice, E2 drives uterine epithelial proliferation, and P4 counteracts E2-induced epithelial proliferation while promoting stromal cell proliferation. E2 is the motivator behind embryonic implantation, whereas E2 and P4 synergistically contribute to decidualization. Estrogen plays its function through activating ER during peri-implantation. ERα, the dominant subtype of ER in mouse uterus, could bind to the specific DNA sequences and exert the transcriptional regulation for target genes, such as LIF and STAT3 that are critical for embryo implantation [35]. ERα knockout mice show compromised implantation with uterine hypoplasia [36]. In mice, it has been revealed that OPN expression in the uterine endometrium is dependent on estrogen [18]. Our results from ovariectomized mouse show that uterine OPN expression is up-regulated by estrogen or by the combination of estrogen and progesterone. ICI182,780 is an antagonist that competes with estrogen for ERα binding, and the inhibition of OPN expression in mice injected with ICI182,780 suggests that E2 regulates endometrial OPN expression via ERα. Previous results verified that E2-induced OPN expression could regulate blastocyst adhesion through activating focal adhesion kinase (FAK) and phosphatidylinositol 3-kinase (PI3K) signaling pathways [17]. However, estrogen has no obvious effect on OPN expression in isolated primary mESCs. Progesterone is tightly related to endometrial stromal cell proliferation and differentiation, and progesterone receptor (PR) knockout mice manifest compromised decidualization; thus, progesterone is indispensable for mouse decidualization. In this study, progesterone induced OPN expression in mESCs but was abrogated by PR antagonists, suggesting that progesterone could regulate OPN expression in the stromal cells through PR. OPN positive macrophages in endometrial stroma contribute to tissue remodeling and trophoblast invasion [18]. About half of OPN deficient mice showed pregnancy failure with abnormal placentation or other mid-gestation dysfunction [19]. Additionally, uterine natural killer (uNK)-originated OPN expression is regulated by progesterone and IL-15 signal pathway, which may play a key role in uNK function [37]. Therefore, progesterone induced OPN expression in stromal cells may play an important role in pregnancy maintenance through involving in cell-ECM interaction, maternal-embryo interaction, immune privilege and placentation.

In mammals, pregnancy initiation and maintenance requires the synthesis and secretion of histotrophs by the endometrial glandular epithelium, which support embryonic development for at least the first trimester of pregnancy. Among the secreted elements are LIF [38], insulin-like growth factor 1 binding protein (IGFBP-1) and glycodelin [39]. In mice, uterine gland knockout results in defective implantation, decidualization and pregnancy failure [40]. Previous studies have identified that OPN is one of the more commonly secreted histotrophs in the uterine flushing fluids from humans and many domestic animals during early pregnancy [41]. Our previous results showed that OPN protein localization in mouse blastocysts is essential for blastocyst-uterine interaction [42]. In this study, immunohistochemistry showed that the OPN protein was mainly localized in the GE on day 4; furthermore, western blot results demonstrated that the OPN protein was found in the uterine flushing fluids collected on days 4 and 5. As an endometrium-originating histotroph, OPN may participate in embryonic development and implantation during peri-implantation. In this study, mouse blastocysts were cultured *in vitro*, with or without different concentrations of rOPN or anti-OPN antibodies. The results showed that rOPN was able to promote the hatching of blastocysts, whereas anti-OPN antibodies could partially inhibit the blastocysts' hatching. These results illustrated that OPN is synthesized by the endometrial glandular epithelium and is secreted into the uterine cavity on day 4, a process that is potentially associated with the progression of blastocyst hatching.

### OPN function in blastocyst activation, adhesion and invasion

Embryo implantation starts with attachment and adhesion between the blastocyst and uterine LE, and it has been generally accepted that ECM proteins contribute to blastocyst adhesion and trophoblast outgrowth [43]. This cell-cell interaction is mediated by the RGD motif that exists in ECM proteins after combining with integrins [44]. The OPN-αvβ3 complex is an acknowledged marker of the implantation window [45]. Our results showed that both OPN mRNA and proteins were up-regulated on day 4 of pregnancy, suggesting that OPN may be involved in the blastocyst adhesion process. Our *in vitro* adhesion assay confirmed that rOPN was able to facilitate the adhesion of blastocysts to FN pre-coated plates, a process that was inhibited by supplementation with soluble anti-OPN antibodies. OPN is characterized by RGD integrin-binding tripeptide and N-linked oligosaccharide motifs, which are able to bind to integrin ligands and mediate cell adhesion, invasion and metastasis. In our *in vitro* adhesion assay, the adhesion rate was decreased by adding soluble RGD peptides, suggesting that OPN activates blastocyst adhesion competency via its RGD sequence. The researches on OPN deficient mice suggested that OPN may not influence the embryo-uterine attachment due to the implantation rates are equivalent between OPN deficient and wild-type mice [19]. Our results showed that secreted OPN protein is concentrated in uterine flushing fluids and functioned in blastocyst hatching, adhesion and invasion in vitro. The discrepancy of OPN function *in vivo* and *in vitro* indicated that other *in vivo*-derived factors could compensate for OPN activation of blastocyst adhesion.

As the embryo attaches to the uterine LE, the trophoblast will invade the stroma, causing the proliferation and differentiation of endometrial stromal cells to form an extensive and well-controlled decidua and the subsequent placenta [46]. The OPN protein is expressed at high levels in the decidua, invading cytotrophoblasts and placenta in humans and mice [31]. Our results showed that both OPN mRNA and proteins were up-regulated at the implantation sites on days 5 and 8 compared with inter-implantation sites. A previous study also showed that OPN is highly expressed in the mouse decidua during the progression of decidualization, although the presence of active blastocysts was required [37]. On day 8 of pregnancy, the OPN protein was localized in the mesometrial region of the decidua and in the anti-mesometrial region of the vascular endothelial cells, suggesting that OPN is expressed in decidual cells and is involved in decidualization and placentation.

In our *in vitro* co-culture model, mouse endometrial stromal cells were pretreated with OPN-targeting siRNA to down-regulate OPN expression. Our results showed that knockdown of OPN was able to inhibit the trophoblast-spreading area in the mESCs. During decidualization, the stroma undergoes comprehensive ECM degradation and reconstruction, a process that relies on the balance of MMPs and TIMPs [47], [48]. The matrix metalloproteinases are a group of zinc-containing, extracellular matrix proteases that play important roles in tissue remodeling, inflammation, fibrosis, and activation of various latent cytokines and cell adhesion molecules. Trophoblasts are able to express MMPs, primarily MMP-9, and other plasminogen activators as a means of degrading ECM components and, consequently, of promoting cell invasion [49], [50], whereas TIMPs are expressed by decidual cells immediately adjacent to the trophoblast [51]. Our experiments showed that the trophoblast expression of MMP-9 is inhibited in OPN-targeted siRNA pretreated mESCs. The expression of OPN has often been found to be related with the presence of MMPs, and OPN is one of the substrates of MMPs [52]. Previous studies have revealed that OPN contributes to the increased levels of MMP-9 in the cardiac and skeletal muscles of mice [53]. Thus, decidua-originating OPN could feasibly increase MMP-9 expression in trophoblasts. Indeed, defects in OPN expression may cause an insufficient expression of MMP-9 in trophoblasts to the point of impaired invasive competency.

**Figure 8:**
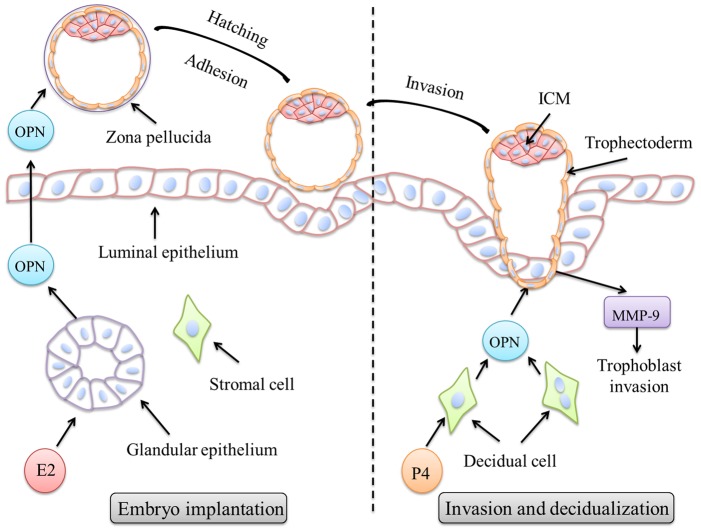
Proposed schematic diagram for OPN roles in mouse embryo implantation and decidualization. Mouse blastocysts entering the uterus and hatching from the zona pellucida are key steps for embryo activation and depend on the synchronization of embryo development and uterine receptivity. The ovarian estrogen (E2) surge induces OPN expression in the glandular epithelium, and OPN proteins are then secreted into uterine cavity to promote blastocyst hatching and adhesion to the luminal epithelium during implantation. After the blastocyst implants into uterine endometrium, uterine stromal cells undergo decidualization, which is characterized by extensive proliferation and differentiation. OPN is highly expressed in decidual cells and regulated by progesterone (P4). Hypothetically, OPN should be able to promote trophoblast cell invasion by regulating the expression and enzymatic activity of MMP-9. In conclusion, OPN expression during peri-implantation and decidualization may contribute to embryo activation and invasion in mice.

In this study, we showed that OPN was expressed in the uterine GE and secreted into the uterine cavity, indicating that OPN may play a role in blastocyst hatching and adhesion. OPN was strongly expressed in the decidua on day 8, and a knockdown of OPN in the mESCs was able to impede the invasion and outgrowth of trophoblast. These data confirm that OPN is involved in trophoblast invasion and in the decidua remodeling process via its regulation of the expression of MMP-9 in trophoblasts. In conclusion, OPN, as an important secreted protein, may be essential for successful embryo implantation and decidualization (Fig. 8).

## Supporting Information

Figure S1 (A) Negative control for Fig. 1B. (B) Negative control for Fig. 7A. (C) Negative control for Fig. 7B. The arrow indicates adhesive blastocyst.
(TIF)
