# Supplementary figures and images for: Osteopontin Is Expressed in the Mouse Uterus during Early Pregnancy and Promotes Mouse Blastocyst Attachment and Invasion In Vitro
Source: PLoS One. 2014 Aug 18;9(8):e104955. doi: 10.1371/journal.pone.0104955 (PMC4136843; doi:10.1371/journal.pone.0104955)

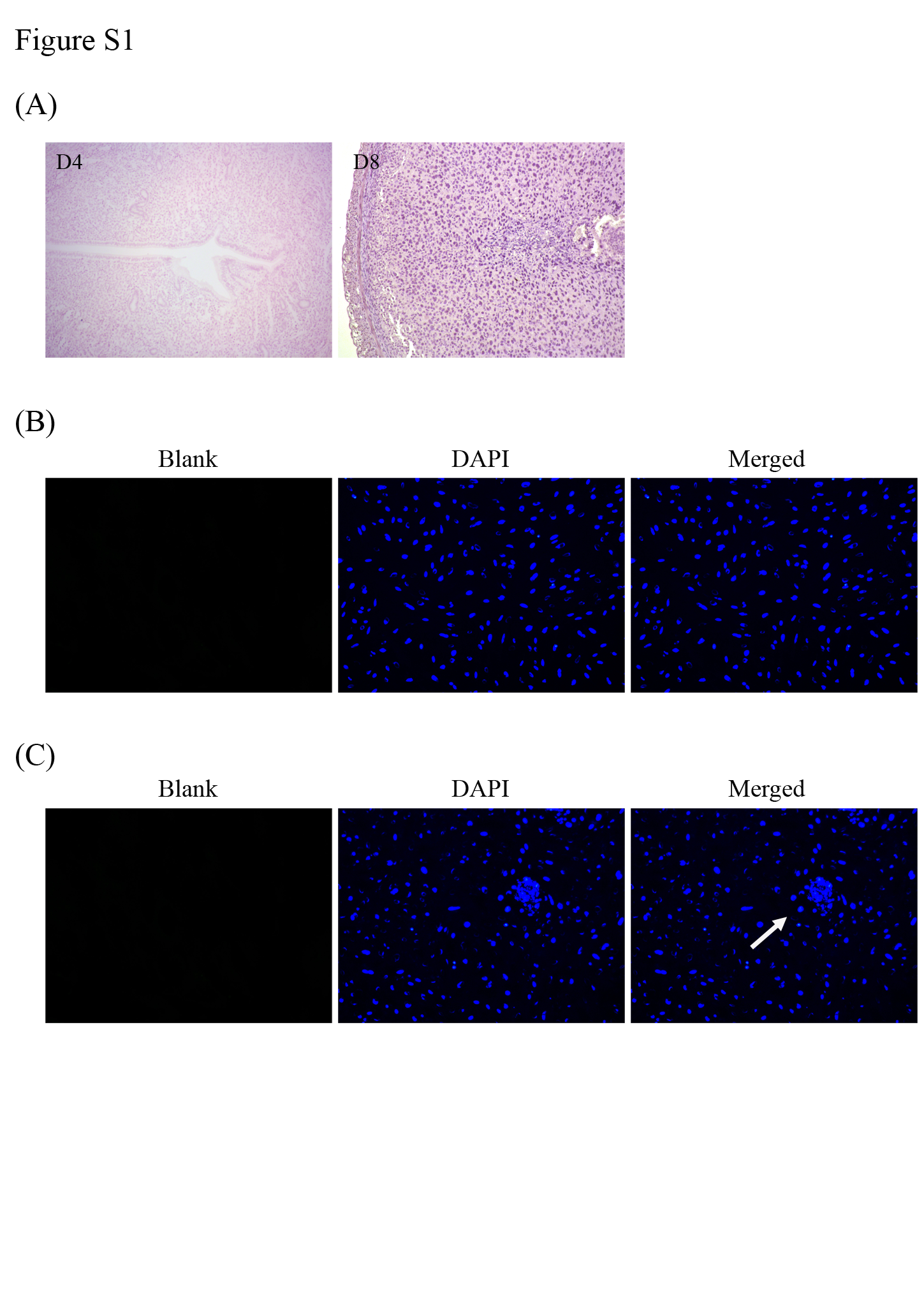

Supplement: Figure S1 — (A) Negative control for Fig. 1B. (B) Negative control for Fig. 7A. (C) Negative control for Fig. 7B. The arrow indicates adhesive blastocyst. (TIF) [file pone.0104955.s001.tif]
